# Machine Learning-Based 5G-and-Beyond Channel Estimation for MIMO-OFDM Communication Systems

**DOI:** 10.3390/s21144861

**Published:** 2021-07-16

**Authors:** Ha An Le, Trinh Van Chien, Tien Hoa Nguyen, Hyunseung Choo, Van Duc Nguyen

**Affiliations:** 1 School of Electronics and Telecommunications, Hanoi University of Science and Technology, Hanoi 100000, Vietnam; anleha1997@gmail.com (H.A.L.); hoa.nguyentien@hust.edu.vn (T.H.N.); 2 School of Information and Communication Technology, Hanoi University of Science and Technology, Hanoi 100000, Vietnam; trinhchien.dt3@gmail.com; 3 Interdisciplinary Centre for Security, Reliability and Trust (SnT), University of Luxembourg, L-1855 Luxembourg, Luxembourg; 4 College of Computing, Sungkyunkwan University (SKKU), Seoul 08826, Korea; choo@skku.edu

**Keywords:** machine learning, channel estimation, MIMO-OFDM, frequency selective channels

## Abstract

Channel estimation plays a critical role in the system performance of wireless networks. In addition, deep learning has demonstrated significant improvements in enhancing the communication reliability and reducing the computational complexity of 5G-and-beyond networks. Even though least squares (LS) estimation is popularly used to obtain channel estimates due to its low cost without any prior statistical information regarding the channel, this method has relatively high estimation error. This paper proposes a new channel estimation architecture with the assistance of deep learning in order to improve the channel estimation obtained by the LS approach. Our goal is achieved by utilizing a MIMO (multiple-input multiple-output) system with a multi-path channel profile for simulations in 5G-and-beyond networks under the level of mobility expressed by the Doppler effects. The system model is constructed for an arbitrary number of transceiver antennas, while the machine learning module is generalized in the sense that an arbitrary neural network architecture can be exploited. Numerical results demonstrate the superiority of the proposed deep learning-based channel estimation framework over the other traditional channel estimation methods popularly used in previous works. In addition, bidirectional long short-term memory offers the best channel estimation quality and the lowest bit error ratio among the considered artificial neural network architectures.

## 1. Introduction

The exponential increases in wireless throughput for many different types of users with high quality of service demands have been predicted to continue in upcoming years [1]. Fifth-generation (5G) and beyond wireless communication has been developed by integrating several disruptive technologies such as Massive MIMO, mmWave communications, and reconfigurable intelligent surfaces to handle the fast growth in wireless data traffic and reliability communications [2,3,4]. The orthogonal frequency division multiplexing (OFDM) technique has been verified to be a contributor due to its inevitable successes in wide-band communication networks. In fact, OFDM is still deployed in 5G systems to combat the frequency selective fading effects, therefore offering good communication quality in multi-path propagation environments [5]. Specifically, the OFDM technique increases the spectrum efficiency significantly compared with a single-carrier approach. When the transmitted signals propagate through the wireless multi-path channels, they are distorted by many detrimental effects; for example, large obstacles, multi-path propagation, local scattering, and mutual interference by sharing the same time and frequency radio resources. To decode the desired signal effectively, the channel state information and its effects should be estimated and compensated at the receiver. For this purpose, the pilot signals should be known to both the transmitter and receiver, which are exploited to perform the channel estimation. In a 5G system, the structure of the pilot symbols in each data frame could be varied depending on the different use cases in practice [6]. We note that, among the traditional channel estimation methods, least squares (LS) estimation is well-known as a low computational complexity method because this estimation requires no prior channel statistics [7,8]. However, LS estimation provides relatively high channel estimation errors in many practical applications, especially for multi-path channels. As an alternative solution, minimum mean square error (MMSE) estimation yields much better channel estimation quality than LS estimation by minimizing the channel estimation errors on average [9]. The closed-form expression of the channel estimates obtained by the MMSE estimation relies on the assumption that, for instance, the propagation channels are modeled by a linear system, while each channel response follows a circularly symmetric complex Gaussian distribution [10,11]. Nonetheless, the MMSE estimation usually has high computational complexity since channel statistic information—i.e., the mean values and the covariance matrices of the propagation channels—is required. In many propagation environments, this statistical information is either extremely difficult to obtain or varies quickly in a short coherence time, making MMSE estimation challenging to implement [12,13].

Machine learning has recently attracted a great deal of attention in both academia and industry for various applications of wireless communications, such as radio resource allocation, physical security, signal decoding, and channel estimation [14,15,16,17,18]. Regarding the channel estimation application, the authors in [19] reported the use of a trained deep neural network (DNN) model with the help of a pilot signal to estimate underwater channels in an efficient manner. In [20], the authors suggested to exploit the channel correlation in both time and frequency domains with a DNN model to perform channel estimation for the IEEE 802.11p standard. Furthermore, in [21], the authors investigated the effects of the channel estimation phase for a wireless energy transfer system and demonstrated that downlink channel estimation is necessary to harvest energy feedback information. In the considered system, a DNN structure makes better channel estimates than the traditional estimations comprising the LS estimation and the linear MMSE (LMMSE) estimation. We emphasize that several sophisticated techniques have been applied to estimate channel state information (CSI) to date. In a MIMO system, we could assume in practice that the CSI from each antenna at the BS shares the same autocorrelation pattern for enhancing the channel estimation quality of a particular terminal [22]. By effectively deploying this property and arranging the CSI from the multiple antennas into a matrix, the system can exploit a well-known technique from the fields of image recognition and image denoising [15,23,24,25] to predict the pattern of CSI variation by means of the channel structure. In particular, a convolutional neural network (CNN) is applied in [26] for channel estimation in a mmWave Massive MIMO system to reduce noise from the estimated channel, thus outperforming the traditional counterparts. In [27], the authors proposed a CNN-based scheme to predict channels in a large-scale MIMO system as the channels age. The authors in [28] used a deep CNN to enhance the channel estimation quality while retaining high performance compared to the traditional methods by utilizing less pilot overhead. The numerical results showed that the data-driven method remarkably improved the prediction quality. However, the authors in those papers did not consider the influences of Doppler frequencies, which can cause significant changes in the channels over time and even make the channels nonstationary. In addition, the velocity of the receiver may often vary; thus, it is important to evaluate the effect of the mismatch of the Doppler frequency between the training and testing stages of a DNN model. Another approach is to treat instantaneous channels as a time series data and then consider the CSI estimation as a typical time series learning problem to model the problem. In this case, there exist several powerful architectures in the literature that can track the long-term correlation of the channel profile effectively, including long short-term memory (LSTM) [29] and the gated recurrent unit (GRU) [30]. The authors in [31] suggested a scheme that integrates an LSTM network and a feed-forward neural network (FNN) in a unified structure to track time-varying channels, but without mobility. Apart from this, the authors in [32] reported the use of a bidirectional GRU network to estimate time-selective fading channels. Because of the ability to learn and predict the relationship among the various realizations of the propagation channels, those recurrent neural network structures showed unprecedented improvements over the traditional suboptimal channel estimation methods. Nonetheless, in both papers, the authors only considered channel estimation in SISO systems. Since MIMO technology has been widely used in many modern wireless communication systems, the evaluation of the use of a recurrent neural network for estimating channel information under the Doppler effect is necessary.

In this paper, we extend our preliminary work [6], which only used a fully-connected deep neural network (FDNN) model to enhance the channel estimation of a MIMO-OFDM system over frequency-selective fading channels. We show the system performance of the proposed deep learning-based channel estimation framework with different receiver velocities and different neural network structures. The channel parameters in each scenario are generated based on the tapped delay line type C model (TDL-C) that was reported by 3GPP [33]. Our main contributions are summarized as follows:

- We construct a MIMO-OFDM system with the channel profile suggested by 3GPP for 5G-and-beyond systems, accounting for the effects of mobility and frequency selective fading. We make a practical assumption that the receiver does not know the instantaneous channels and that the transmitted data symbols should include pilot signals for the channel estimation;
- We propose a general deep neural network that assists with the traditional channel estimation technique. Our framework does not require any prior information of channel statistics. In particular, the proposed deep learning-based channel estimation framework exploits a neural network to learn the features of the actual channels by utilizing the channel estimates obtained from the LS estimation as the input;
- We provide three examples of exploiting DNN structures: a fully connected DNN, CNN, and bi-LSTM. With these typical examples, we evaluate the degree to which the system performance is improved by the assistance of a DNN in comparison to the LS estimation;
- We evaluate the performance of the DNN-based channel estimation framework by extensive numerical results and show its effectiveness by comparing it with the traditional LS estimation and LMMSE estimation, in terms of both the mean square error (MSE) and bit error rate (BER). We further analyze whether the proposed estimation is robust to Doppler effects.

This paper is organized as follows: Section 2 presents in detail the considered MIMO-OFDM system for the 5G-and-beyond channel profile. The deep learning framework that enhances the channel estimation quality is presented in Section 3 with the three popular neural network structures. The computational complexity of the proposed framework is also analyzed in this section. The extensive simulations used to verify the machine learning-based channel estimation are shown in Section 4 with different setups. Finally, Section 5 presents the conclusions of the paper.

*Notation*: The upper and lower-case bold letters are used to denote the matrices and vectors, respectively. The notation CN(·,·) denotes the circularly symmetric Gaussian distribution and C is the complex field. The notation E{·} is the expectation of a random variable. The notation ⊗ is the convolutional operator, while ⊙ is the Hadamard product. O(·) is the big-O notation that expresses the order of computational complexity. Finally, ${∥\cdot ∥}_{2}$ and ${∥\cdot ∥}_{F}$ denote the Euclidean of a vector and the Frobenius norm of a matrix, respectively.

## 2. System Model

**Figure 1:**
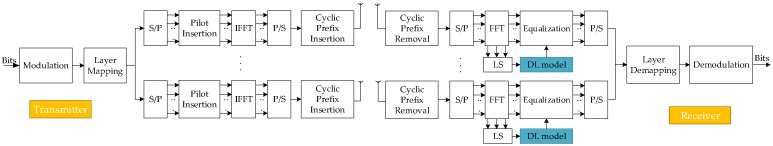
The illustration of the considered MIMO-OFDM system model with the proposed DNN-aided module in blue. In the figure, CP denotes cyclic prefix; S/P denotes serial to parallel; P/S denotes parallel to serial; IFFT denotes inverse fast Fourier transform; and FFT denotes fast Fourier transform.

In this section, we present a MIMO-OFDM system that comprises a transmitter sending signals to a receiver as illustrated in Figure 1. The transmitter and receiver antenna arrays have $N_{T}$ and $N_{R}$ antennas, respectively, therefore creating an $N_{T}\times N_{R}$ MIMO channel model that is modeled by the 5G channel profile.

### 2.1. Transmitter

At the transmitter side, the binary data are first encoded and mapped with quadrature amplitude modulation (QAM) by utilizing the modulation block. We suppose that the system transmits data in *T* time slots, and the QAM symbols at time slot *t*, t=1,⋯,T, are combined to a data vector $x\left(t\right)\in C^{N}$ as

(1) $$x\left(t\right)=[x_{1}\left(t\right),x_{2}\left(t\right),\cdots ,x_{N}\left(t\right)],$$

where *N* is the total number of modulation symbols. The encoded data are then separated into the $N_{T}$ vectors corresponding to the $N_{T}$ transmit antennas as follows:

(2) $$x_{i}\left(t\right)=\left[x_{i}\left(t\right),x_{i+N_{T}}\left(t\right),x_{i+2N_{T}}\left(t\right),\cdots \right]\;i=1,2,\cdots ,N_{T}.$$

The data for each antenna are converted from serial to parallel, and then the pilot signals, which are known from both the transmitter and receiver, are inserted along with data in every layer for channel estimation purposes. We denote $x_{a}\left(t\right)$ with $a=1,\cdots ,N_{T}$ being the signal vector with a pilot inserted into the corresponding data $x_{i}\left(t\right)$; then, the IFFT (inverse fast Fourier transform) block is applied to $x_{a}\left(t\right)$ such that the signals are transformed from the frequency domain into the time domain (denoted by ${\tilde{x}}_{a}\left(t\right)$) as

(3) $$\begin{array}{c} {\tilde{x}}_{a}\left(t\right)=\mathrm{IFFT}\left\{x_{a}\left(t\right)\right\}. \end{array}$$

After that, the cyclic prefix (CP) with the length $N_{G}$ is inserted as a guard interval to alleviate the ISI (inter-symbol interference) by utilizing the CP insertion block. By including the cyclic prefix, the transmitted signal that is denoted by ${\tilde{x}}_{ga}\left(t\right)$ is formulated in the time domain as follows:

(4) $${\left[{\tilde{x}}_{ga}\left(t\right)\right]}_{n}=\left\{\begin{array}{cc} {\left[{\tilde{x}}_{a}\left(t\right)\right]}_{n+N_{\mathrm{FFT}}} & n=-N_{G},-N_{G}+1,\ldots ,-1\\ {\tilde{x}}_{a}{\left(t\right)]}_{n} & n=0,1,\ldots ,N_{\mathrm{FFT}}-1, \end{array}\right.$$

where $N_{\mathrm{FFT}}$ is the FFT size. This means that the last $N_{G}$ samples of ${\tilde{x}}_{a}\left(t\right)$ are used as a cyclic prefix and inserted into the beginning of this symbol, resulting in the signal ${\tilde{x}}_{ga}\left(t\right)$ with a length of $N_{\mathrm{FFT}}+N_{G}$.

### 2.2. 5G-and-Beyond Channel Model

In this paper, we consider the 5G-and-beyond channel model, which is defined by the 3GPP standard in [33]. The 5G-and-beyond channel model includes the effect of multi-path and Doppler shifting, which cause frequency-selective fading and time-selective fading, respectively. In particular, we exploit the TDL-C model defined for the NLOS channel for the full frequency range from 0.5 GHz to 100 GHz [33] with Rayleigh fading distribution. The Doppler spectrum of each tap is characterized by a classical Jake’s spectrum shape, which is expressed as

(5) $$S\left(f\right)=\frac{1}{\pi f_{d}\sqrt{1-{\left(\frac{f}{f_{d}}\right)}^{2}}},\;\left|f\right|<f_{d},$$

where $f_{d}$ (Hz) is the maximum Doppler shift; i.e., $f_{d}=\frac{vf_{c}}{c}$, for a given speed *v*(m/s) and a carrier frequency $f_{c}$(Hz), with $c\approx 3\times 10^{8}$ being the light speed. The auto-correlation of Jake’s Doppler spectrum is [34]

(6) $$R\left(\tau \right)=\int_{-f_{d}}^{f_{d}}S\left(f\right)e^{2\pi \tau }df=J_{0}\left(2\pi f_{d}\tau \right),$$

where $J_{0}\left(.\right)$ is the first kind of Bessel function of order 0. From the continuous form in (6), the discrete form of the auto-correlation function is defined as follows:

(7) $$R\left[l\right]=J_{0}(2\pi f_{d}\left|l\right|T_{sym}),$$

where *l* and $T_{sym}$ are the symbol index and the symbol duration, respectively. We denote $h_{a,b}\left(\tau_{i},t\right)$ as the time-variant channel impulse response from the *a*-th transmission antenna ($a=1,\cdots ,N_{T}$) to the *b*-th receiver antenna ($b=1,\cdots ,N_{R}$), where $\tau_{l}$ is the transmission delay at the *l*-th tap of the propagation channels. A mathematical description of the frequency-selective and time-variant channel model is given in [35] as follows

(8) $$h_{a,b}\left(\tau_{l},t\right)=\sum_{l=0}^{L-1}h_{l}\delta \left(\tau_{l}-t\right)\times \exp j[2\pi f_{D,l}\left(t-\tau_{l}\right)-2\pi f_{c}\tau_{l}],$$

with *l* is the index of taps, $h_{l}$ represents the *l*-th resolved amplitude, and $\tau_{l}$ represents the express delay of the *l*-th tap. $f_{D,l}=v\left(t\right)f_{c}\cos\left[\theta_{l}\right]/c$ is the Doppler frequency induced by the relative movement of the Tx and Rx, v(t) represents the relative velocity, $\theta_{l}$ denotes the aggregate phase angle of all components arriving in the *l*-th tap, and *c* is the speed of light.

**Figure 2:**
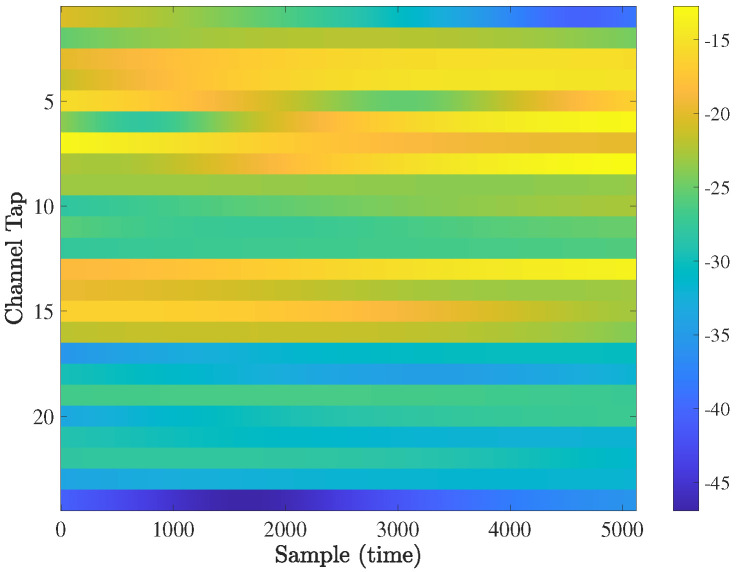
The 2 time—varying channel profile with $f_{d}=200$ Hz in the 20 OFDM symbols.

**Figure 3:**
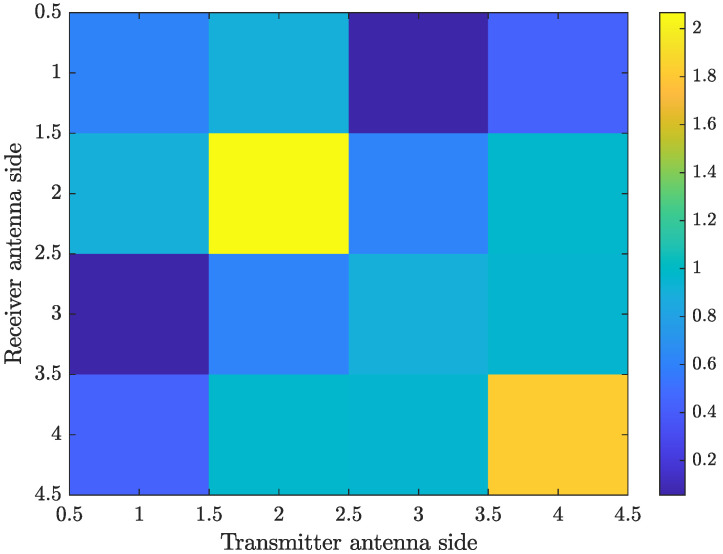
The expectation $E\{HH^{H}\}$, where $H\in C^{N_{T}\times N_{R}}$ is the channel matrix of a subcarrier. Here, $N_{T}=N_{R}=4$ and $f_{d}=200$ Hz.

To model the propagation channels in this paper, we exploit the Matlab 5G toolbox [33] to simulate the instantaneous channels. The 5G-and-beyond channels have the TDL-C profile displayed in Figure 2, with the color-map displaying the channel gain. In more detail, the channel gain varies from −12 dB to −47 dB. This figure indicates that the considered channel profile is not sparse, which is a consequence of the mobile communication carrier frequency at sub-6GHz; i.e., here, the carrier frequency is set to 4 GHz (the channel estimation quality can be enhanced if a proper domain, in which the channels are spares, is determined, and thus a sparse channel estimation technique is effectively utilized. This work is left for the future). In addition, Figure 3 plots the expectation $E\{HH^{H}\}$, where H is the channel matrix of a subcarrier. It shows that all the coefficients are non-zero, therefore verifying the spatial correlation among the channels.

By utilizing the channel model in (8) and the transmitted signal in (4), the received signal after passing through the 5G multi-path channel is formulated as

(9) $${\tilde{y}}_{gb}\left(t\right)=\sum_{a=1}^{N_{T}}{\tilde{h}}_{a,b}\left(\tau ,t\right)⊗{\tilde{x}}_{ga}\left(t\right)+{\tilde{n}}_{b}\left(t\right),$$

where ${\tilde{h}}_{a,b}\left(\tau ,t\right)=\left[h_{a,b}\left(\tau_{1},t\right),\ldots ,h_{a,b}\left(\tau_{L},t\right)\right]$; ${\tilde{n}}_{b}\left(t\right)$ is the additive noise vector, whose elements are independent and identically distributed random variables following a circularly symmetric complex Gaussian distribution with zero-mean and variance $\sigma_{n}^{2}$. From the received signal in Equation (9), we are able to estimate the propagation channels and analyze the system performance as shown below.

### 2.3. Receiver

At the receiver side, the cyclic prefix is first removed from the received signal ${\tilde{y}}_{gb}\left(t\right)$ on each antenna using the cyclic prefix removal module to obtain the vector ${\tilde{y}}_{b}\left(t\right)$ of the length $N_{\mathrm{FFT}}$. The signal is then converted to the parallel form and transformed into the frequency domain by the FFT block, which gives a frequency domain signal $y_{b}\left(t\right)$ of

(10) $$y_{b}\left(t\right)=\mathrm{FFT}\left\{{\tilde{y}}_{b}\left(t\right)\right\}.$$

The pilot signal is exacted from the frequency-domain signal for channel estimation purposes. After estimating the channel, the received signal $y_{b}\left(t\right)$ is equalized and congregated into a serial sequence from all the receiver antennas by the layer demapping module. The signal is then demodulated by the demodulation scheme, which corresponds to the approach used by the transmitter. At this point, the output of the MIMO-OFDM system model is obtained as the final binary data sequence.

### 2.4. 5G Pilot Structure

In 5G wireless communication systems, the demodulation reference signals (DM-RS) are used as pilots to facilitate channel estimation. DM-RS signals are generated based on a sequence defined in the 3GPP standard [36] as

(11) $$r\left(n\right)=\frac{1}{\sqrt{2}}\left[1-2c\left(2n\right)\right]+j\frac{1}{\sqrt{2}}\left[1-2c\left(2n+1\right)\right],$$

where c(i) is the pseudo-random sequence and is defined by a length-31 Gold sequence as [36]

(12) $$\begin{array}{cc} c(n) & =[x_{1}\left(n+1600\right)+x_{2}\left(n+1600\right)]mod2 \end{array}$$

(13) $$\begin{array}{cc} x_{1}\left(n+31\right) & =[x_{1}\left(n+3\right)+x_{1}\left(n\right)]mod2 \end{array}$$

(14) $$\begin{array}{cc} x_{2}\left(n+31\right) & =[x_{2}\left(n+3\right)+x_{2}\left(n+2\right)+x_{2}\left(n+1\right)+x_{2}\left(n\right)]mod2, \end{array}$$

where mod is the modulo operator, and the 31-first sequence $x_{1}\left(n\right)$ and $x_{2}\left(n\right)$ are initialized as

(15) $$\begin{array}{cc} x_{1}\left(n\right) & =\left\{\begin{array}{c} 1,\;n=0\\ 0,\;n=1,2,\cdots ,30 \end{array}\right. \end{array}$$

(16) $$\begin{array}{cc} c_{init} & =\;\sum_{n=0}^{30}x_{2}\left(n\right)2^{n}. \end{array}$$

In the initialization of sequence $x_{2}\left(n\right)$, the value of $c_{init}$ depends on the application of the sequence c(n). In the channel estimation application, the value of $c_{init}$ is calculated as [36]

(17) $$c_{init}=\left[2^{17}\left(N_{symb}^{slot}n_{s,f}+l+1\right)\left(2N_{IDSCID}^{n}+1\right)+2N_{ID}^{n_{SCID}}+n_{SCID}\right]mod2^{31},$$

where $N_{symb}^{slot}=14$ is the number of OFDM symbols in slot 1, $n_{s,f}=10$ is the number of slots in frame 1, and *l* is the OFDM symbol index. The parameters $N_{ID}^{0},N_{ID}^{1}\in \left\{0,1,\cdots ,65535\right\}$ and $n_{SCID}\in \left\{0,1\right\}$ are the parameters of the 5G system. In our paper, for simplicity, we set these parameters equal to zero.

**Figure 4:**
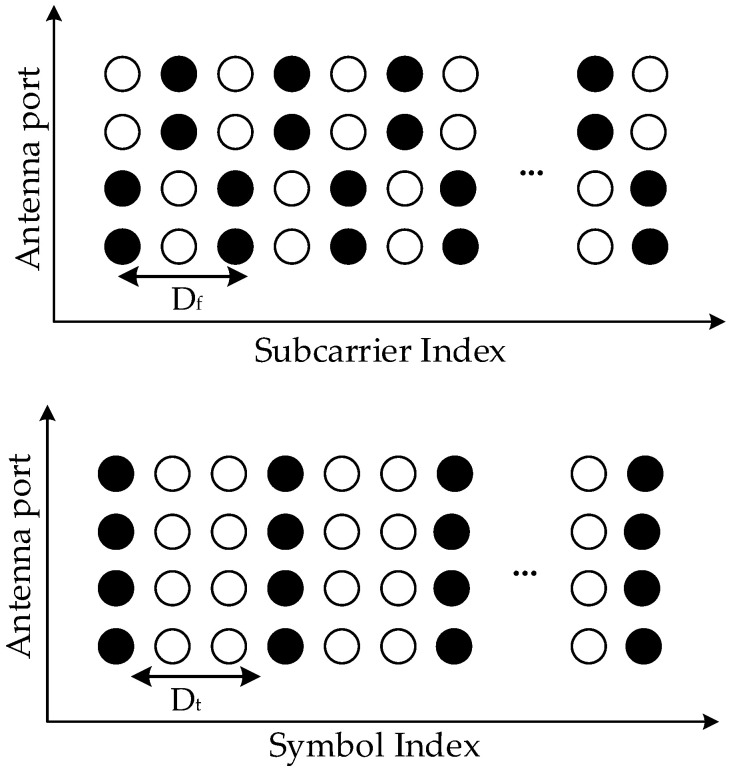
The pilot structure used in the considered MIMO-OFDM system.

The pilot signals are then mapped according to the pilot structure defined in [36]. In 5G systems, the pilots are arranged in a comb type across transmission antennas, as illustrated in Figure 4. The pilot symbols are uniformly spaced in the time domain, denoted by $D_{t}$, and in the frequency domain, denoted by $D_{f}$. The values of $D_{t}$ and $D_{f}$ depend on the different use cases of a 5G system, which are defined explicitly in, for example, [37]. Among transmission antennas, pilot signals are arranged in an alternating way. By applying this design of a pilot pattern into our paper, the pilot signal in each OFDM symbol is calculated as

(18) $$\begin{array}{cc} x_{p}\left(k\right) & =r(n) \end{array}$$

(19) $$\begin{array}{cc} k & =D_{f}n+\Delta \end{array}$$

(20) $$\begin{array}{cc} n & =0,1,\cdots N_{P}, \end{array}$$

where *k* denotes the subcarrier index, $N_{P}=N_{FFT}/D_{f}$ is the number of pilot signals in an OFDM symbol, and Δ defines the pilot position in the frequency domain for each transmission antenna, the value of which can be found in Table 7.4.1.1.2-1 in [36].

## 3. Deep Learning-Based Channel Estimation

In wireless communications systems, coherent detection requires knowledge of the propagation channels between the transmitter and the receiver, which are possible to estimate by utilizing conventional estimation techniques. In this section, we present the two widely-used channel estimation schemes that motivate us to exploit deep learning frameworks to improve the channel estimation errors.

### 3.1. Motivations

As long as no inter-carrier interference occurs, each subcarrier can be expressed as an independent channel, therefore preserving the orthogonality among the subcarriers. The orthogonality allows each subcarrier component of the signal in (10) to be expressed as the Hadamard product of the transmitted signal and channel frequency response at the subcarrier [34] as

(21) $$y_{b}\left(t\right)=\sum_{a=1}^{N_{T}}h_{a,b}\left(t\right)⊙x_{a}\left(t\right)+n_{b}\left(t\right),$$

where $n_{b}\left(t\right)$, $h_{a,b}\left(t\right),$ and $x_{a}\left(t\right)$ are the Fourier transforms of the noise, channel, and signal, respectively (unless we are working in the frequency domain).

Of all the traditional channel estimation methods, LS estimation is one of the most common approaches. We denote by ${\hat{h}}_{\mathrm{LSb}}$ the channel estimate from the transmission antennas at the *b*-th receiver antenna obtained by this estimation method. LS estimation gives the closed-form expression of the channel estimate as [8]

(22) $${\hat{h}}_{\mathrm{LSb}}\left(t\right)={\left([X{\left(t\right)}^{H}X\left(t\right)]\right)}^{-1}X^{H}\left(t\right)y_{b}\left(t\right),$$

where ${\left(\cdot \right)}^{H}$ denotes the Hermitian transpose, and

(23) $$X\left(t\right)={\left[\mathrm{diag}\left(x_{1}\left(t\right)\right),\cdots ,\mathrm{diag}\left(x_{N_{T}}\left(t\right)\right)\right]}^{T}$$

is the $N_{P}\times \left(N_{T}N_{P}\right)$ matrix, denoting the transmitted signal from the transmission antennas; $N_{P}$ is the number of the pilot signals in an OFDM symbol; and ${\left(\cdot \right)}^{T}$ is the regular transpose. The channel estimate from each transmission antenna can be formulated as

(24) $${\hat{h}}_{\mathrm{LSbi}}\left(t\right)={\left[{\left[{\hat{h}}_{\mathrm{LSb}}\left(t\right)\right]}_{\left(i-1\right)N_{P}},\ldots ,{\left[{\hat{h}}_{\mathrm{LSb}}\left(t\right)\right]}_{iN_{P}-1}\right]}^{T},i=1,\cdots ,N_{T}.$$

Then, the channel responses from all sub-carriers can be obtained by applying a linear interpolation method. It should be noted that LS estimation is a widely-used estimation approach because of its simplicity. Nevertheless, this technique does not exploit the side information from noise and statistical channel properties, such as the spatial correlation among antennas, in the estimation, and thus high channel estimation error can occur when applying LS estimation for propagation environments with a high mobility.

To cope with the above drawbacks, one can utilize the LMMSE estimation approach, which minimizes the mean square error. For LMMSE estimation, the channel estimate is formulated in the closed form expression as [34]

(25) $${\hat{h}}_{\mathrm{LMMSEbi}}\left(t\right)=R_{h{\hat{h}}_{\mathrm{LSbi}}}{\left(R_{hh}+\frac{\sigma_{n}^{2}}{\sigma_{x}^{2}}I_{N_{P}}\right)}^{-1}{\hat{h}}_{\mathrm{LSbi}}\left(t\right),\;i=1,\cdots ,N_{T},$$

where ${\hat{h}}_{\mathrm{LMMSEbi}}\left(t\right)$ is the LMMSE estimated channel from the i−th transmission antenna at the b−th receiver antenna, $R_{hh}=E\left\{hh^{H}\right\}$ is the auto-correlation matrix of the channel response in the frequency domain with the size of $N_{P}\times N_{P}$; $R_{h{\hat{h}}_{\mathrm{LSbi}}}=E\left\{h{\hat{h}}_{\mathrm{LSbi}}^{H}\right\}$ is the cross-correlation between the actual channel and the channel estimate obtained by the LS estimation with the size of $N_{\mathrm{FFT}}\times N_{P}$; $\sigma_{x}^{2}$ is the variance of the transmitted signals, respectively; $I_{N_{P}}$ is the identity matrix of size $N_{P}\times N_{P}$. The impacts of both noise and spatial correlation among the antennas are taken into account by LMMSE estimation, which is able to improve the channel estimation accuracy. However, LMMSE estimation requires the prior knowledge of channel statistical properties; thus, the computational complexity is higher than LS estimation. Additionally, since it may be difficult to obtain the exact distribution of channel impulse responses in general [38], the performance of the LMMSE estimation cannot always be guaranteed.

### 3.2. Fully Connected Deep Neural Network-Based Channel Estimation

**Figure 5:**
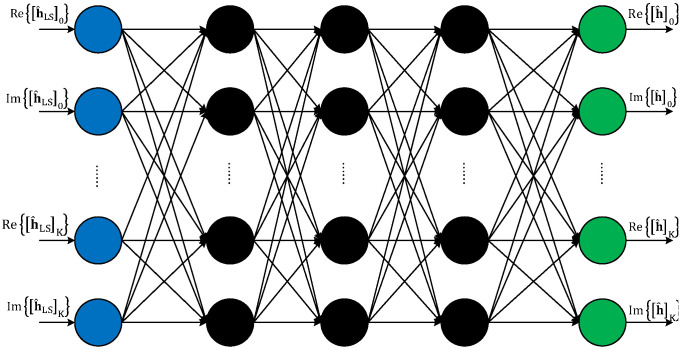
The illustration of the FDNN-based channel estimation.

To overcome the aforementioned drawbacks of LS and LMMSE estimation approaches, we propose a FDNN-aided estimation that minimizes the MSE between the channel estimate obtained by LS estimation and the actual channel. The structure of the proposed FDNN-based channel estimation is depicted in Figure 5. As shown in this figure, the proposed FDNN structure is organized as layers including the input layer, hidden layers, and output layer. Notice that an FDNN may have many hidden layers. However, for the considered MIMO-OFDM system, the proposed FDNN structure is designed with 3 hidden layers that include multiple neurons. In particular, a neuron is a computational unit that performs the following calculation:

(26) $$o=f\left(z\right)=f\left(\sum_{i=1}^{M}w_{i}x_{i}+b\right),$$

where *M* is the number of inputs to the neuron for which $x_{i}$ is the *i*-th input (i=1,…,M); $w_{i}$ is the *i*-th weight corresponding to the *i*-th input; *b* is a bias; and *o* is the output of this neuron. In Equation (26), f(.) is an activation function that is used to characterize the non-linearity of the channel data. In our proposed FDNN-based channel estimation, we borrow the tanh function as the activation function, which is defined as

(27) $$f\left(z\right)=\frac{e^{z}-e^{-z}}{e^{z}+e^{-z}},$$

where *e* is Euler’s number. To minimize the mean square error, the FDNN-based channel estimation is used to learn the actual channel information provided by the channel estimates obtained from the LS estimation as the input. In more detail, we define a realization of the input for the training process as

(28) Mn−FDNN=Reh^LSn(t)0,Imh^LSn(t)0,…,Reh^LSn(t)K,Imh^LSn(t)K,

where ${\hat{h}}_{\mathrm{LS}}^{n}\left(t\right)$ is LS-estimated channel gathered from all received antennas, where the superscript *n* denotes the *n*-th realization; K is the number of channel samples that FDNN can handle; and the Re{·} and Im{·} operators give the real and imaginary part of a complex number, respectively. The output of the neural network is formulated as

(29) On−FDNN=Reh^n(t)0,Imh^n(t)0,…,Reh^n(t)K,Imh^n(t)K,

where ${\hat{h}}^{n}\left(t\right)$ is the output of the neural network at the *n*-th realization. In Equations (28) and (29), we separate the channel estimates into the real and imaginary parts to handle the complex numbers for the use of the FDNN neural network. The learning process handles the one-by-one mapping as

(30) Reh^LSn(t)s,Imh^LSn(t)s→Reh^n(t)s,Imh^n(t)s,s=0,…,K.

As desired, the output of the neural network should be identical to the actual channels. Alternatively, the purpose of the FDNN-aided estimation is to minimize the MSE between the prediction and actual channels on average; thus, the loss function utilized for the training phase is defined as

(31) $$L_{\mathrm{FDNN}}\left(W,B\right)=\frac{1}{NK}\sum_{n=1}^{N}\sum_{t=1}^{T}∥{\hat{h}}^{n}\left(t\right)-h^{n}\left(t\right){∥}_{2}^{2},$$

where *N* is the number of realizations used for training, and $h^{n}\left(t\right)$ is the actual channel corresponding to ${\hat{h}}^{n}\left(t\right)$. W and B include all the weights and biases, respectively. From a set of initial values, the weights and biases are updated by minimizing the loss function (31) with forward and backward propagation [15].

### 3.3. Convolutional Neural Network-Based Channel Estimation

**Figure 6:**
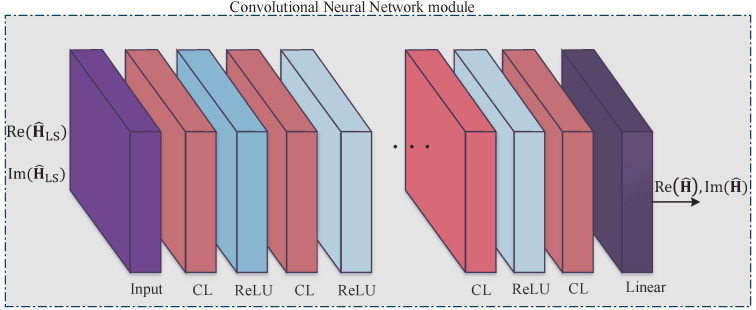
The illustration of the CNN-based channel estimation.

CNN models have been proposed for image denoising algorithms and have been well studied by the image processing community. CNN models can be applied to learn the mapping from noisy images to clean images [39,40], therefore mitigating noise in the images. In addition, due to the sharing of weights and biases, a CNN can reduce the number of parameters, which reduces the complexity of the system. Based on these ideas, we can use CNN to learn the mapping from noisy channels obtained by an LS estimator to the true channels. The structure of the proposed CNN-aided estimation is shown in Figure 6. As depicted in the figure, the proposed CNN consists of a 2D input layer, convolution layers, activation layers, and a linear layer. The 2D input layer takes the LS-estimated channel as an input, which is separated into the real part and image part and reshaped to a 2D matrix form. The channel matrix is then fed to the convolution layers. We denote by L the set of convolution layers for CNN. Each convolution layer l∈L includes $c_{l}$ convolution kernels of size $k_{l}\times k_{l}$ that are convolved with the layer input $I_{l}\in R^{a_{l-1}^{1}\times a_{l-1}^{2}\times c_{l-1}}$, where $a_{l-1}^{1}$ and $a_{l-1}^{2}$ are the size of the (l−1)-th convolution layer. The output of the *l*-th convolution layer $O_{l}\in R^{a_{l}^{1}}\times a_{l}^{2}\times c_{l}$ is

(32) $$O_{l}=\mathrm{Conv}\left(I_{l},w_{l}\right)+b_{l},\;l\in L,$$

where $w_{l}\in R^{k_{l}\times k_{l}\times c_{l}}$ and $b_{l}\in R^{a_{l}^{1}\times a_{l}^{2}\times c_{l}}$ are the weights and biases of the convolution kernel for the *l*-th convolution layer, respectively, and Conv(·,·) is the convolution operator. For the proposed CNN model, after each convolution layer, we apply the well-known rectified linear unit (ReLU) activation layer, which is given as

(33) ReLU(z)=max(0,z).

In particular, to train the CNN model, we first reshape the LS-estimated channel from all antennas into the matrix form ${\hat{H}}_{\mathrm{LS}}^{n}\in C^{N_{T}N_{R}\times N_{FFT}}$, separate it into a real part and image part, and then define a realization of the input for the training process as

(34) Mn−CNN=ReH^LSn,ImH^LSn.

In a similar manner, the corresponding output of the CNN is formulated as

(35) On−CNN=ReH^n,ImH^n,

which contains the real and imaginary matrices of the channel estimates. The CNN model is trained to handle the following matrix mapping as

(36) ReH^LSn,ImH^LSn→ReH^n,ImH^n.

The purpose of applying the CNN model is to minimize the mean square error between the estimated and the true channels. Therefore, we use the loss function, which is defined as follows:

(37) $$L_{\mathrm{CNN}}\left(W,B\right)=\frac{1}{N}\sum_{n=1}^{N}∥{\hat{H}}^{n}-H^{n}{∥}_{F}^{2},$$

where *N* is the number of realizations used for training, and $H^{n}$ is the actual channel in the matrix shape corresponding to ${\hat{H}}^{n}$. W and B include all the weights and biases, respectively. During the training process, the weights and biases of the CNN will be updated by minimizing the loss function (37). We stress that the loss function (37) shares the same training data with that in Equation (31), but the fine structure is different. Specifically, the instantaneous channels are stacked in the vector form in Equation (31), while it is arranged in a matrix form in Equation (37) to make use of the benefits of the CNN.

### 3.4. Long Short-Term Memory-Based Channel Estimation

**Figure 7:**
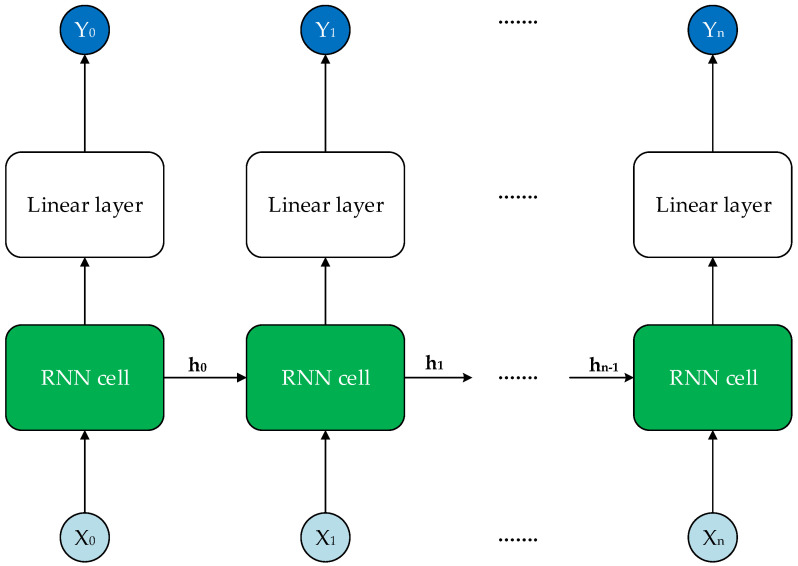
The illustration of the proposed RNN model.

In the two previous subsections, we proposed two deep learning-based channel estimation methods: FDNN-based and CNN-based channel estimation approaches. However, those two methods have no ability to exploit the long-term correlation of the channels, and thus they could not reach the optimal performance in general. To address this issue, one good choice is to apply a neural network that has the ability to study the behaviors of the channel correlations, such as a recurrent neural network (RNN). The simple structure of a one-layer RNN is given in Figure 7. As we can see from this figure, the input of the RNN cell in the current time step is the output of the RNN cell in the previous time step. Working in this way, the RNN can remember the past information of the input. The basic RNN cell is the computation unit, which performs the following calculation [41]:

(38) $$\begin{array}{cc} \; & h_{t}=f\left(W_{ih}x_{t}+b_{ih}+W_{hh}h_{t-1}+b_{hh}\right), \end{array}$$

(39) $$\begin{array}{cc} \; & Y_{t}=f\left(W_{ho}h_{t}+b_{ho}\right), \end{array}$$

where f(·) is the activation function; $h_{t}$ and $h_{t-1}$ are the hidden states at the time step *t* and t−1, respectively; $x_{t}$ and $Y_{t}$ are the input and the output at the time step *t*; $W_{ih}$,$W_{hh}$, and $W_{ho}$ are the weights for the input layer to the hidden layer, the hidden layer to the next hidden layer, and the hidden layer to the output layer, respectively; and $b_{ih}$, $b_{hh}$, and $b_{ho}$ are the corresponding biases.

However, the simple RNN cell has several weaknesses: first, it has no ability to exploit the future information of the data, while the channel at the time step *t* has a relation not only with the past but also the future. Thus, the bidirectional network should be used in this case to obtain better performance. Second, another problem with using a simple RNN cell is that it cannot capture long-term information. One solution for this problem is to use LSTM instead. Consequently, in this paper, we propose a bidirectional-long short-term memory (bi-LSTM) network for 5G channel estimation to overcome the above-mentioned weaknesses.

**Figure 8:**
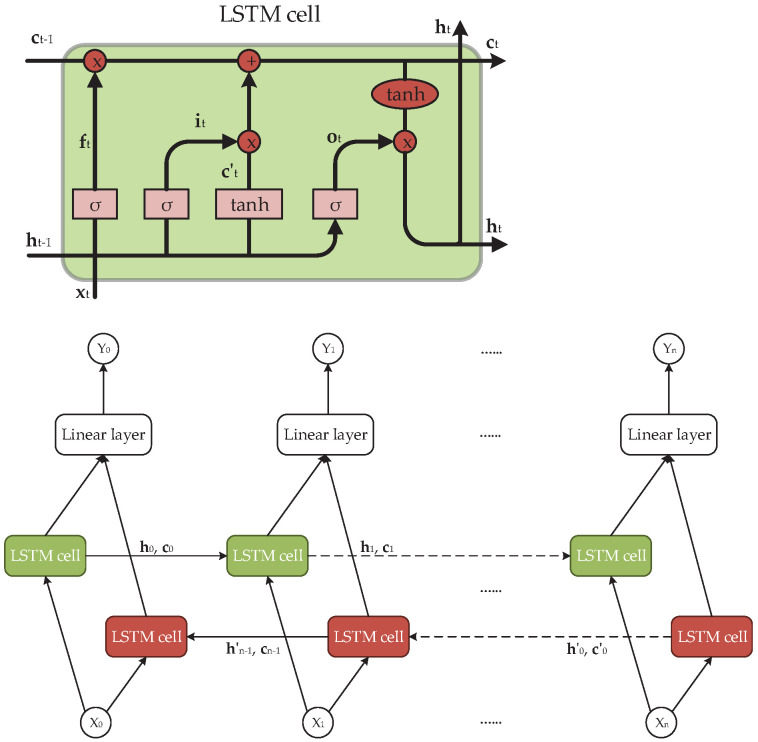
The structure of an LSTM cell (**top**) and the structure of the proposed bi-LSTM approach (**bottom**).

The structure of the proposed bi-LSTM network for the channel estimation is illustrated in Figure 8. In the bi-LSTM structure, the simple RNN cell is replaced by the corresponding LSTM cell, which has the structure shown in the top of Figure 8. The computation of the LSTM cell will give the result as shown in the following equations [41]:

(40) $$\begin{array}{cc} \; & f_{t}=f\left(W_{f}h_{t-1}+U_{f}X_{t}+b_{f}\right), \end{array}$$

(41) $$\begin{array}{cc} \; & i_{t}=f\left(W_{i}h_{t-1}+U_{i}X_{t}+b_{i}\right), \end{array}$$

(42) $$\begin{array}{cc} \; & c_{t}^{{}^{\prime }}=\tanh\left(W_{c}h_{t-1}+U_{c}X_{t}+b_{c}\right), \end{array}$$

(43) $$\begin{array}{cc} \; & c_{t}=f_{t}⊙c_{t-1}+i_{t}⊙c_{t}^{{}^{\prime }}, \end{array}$$

(44) $$\begin{array}{cc} \; & o_{t}=f\left(W_{o}h_{t-1}+U_{o}X_{t}+b_{o}\right), \end{array}$$

(45) $$\begin{array}{cc} \; & h_{t}=o_{t}⊙\tanh\left(c_{t}\right), \end{array}$$

where tanh is the hyperbolic tangent function, and $W_{f}$, $W_{i}$, $W_{c}$, $W_{o}$, $U_{f}$, $U_{i}$, $U_{c}$, $U_{o}$, $b_{f}$, $b_{i}$, $b_{c}$, and $b_{o}$ are correspondingly the weights of matrices and biases. The forget function $f_{t}$ defines which information will be forgotten by the LSTM cell, $c_{t}$ is the cell state that contains the important information from the past, and $c_{t}^{{}^{\prime }}$ is a new candidate value that defines which information will be updated to the cell state $c_{t}$ and $h_{t}$ is the hidden state function of the LSTM cell. By working in this way, the LSTM cell can capture the important information from the past and avoid the redundant information, thus providing a greater ability to capture the information compared to the simple RNN cell. The bottom of Figure 8 shows the structure of the bi-LSTM network. As we can see, the bi-LSTM approach is the combination of two LSTM networks with two different directions. The output of the bi-LSTM takes the outputs of the two LSTM cells into consideration via the linear layer as

(46) $$Y_{t}=WH_{t}+b,$$

where $H_{t}$ is the hidden state concatenated from the forward hidden state $h_{t}$ and the backward hidden state $h_{t}^{{}^{\prime }}$, and W and b are the weights and biases of the linear layer, respectively. Therefore, the bi-LSTM approach can exploit the relation of both history and the future with the data in the current time step. To apply the bi-LSTM model for our system, we first gather the LS-estimated channels from all antennas and then define a realization of the input for the training process as

(47) Mn−bi−LSTM=Reh^LSn(0);Imh^LSn(0),⋯,Reh^LSn(L−1);Imh^LSn(L−1),

where *L* is the sequence length considered for bi-LSTM network. Note that the input of bi-LSTM ${\hat{h}}_{LS}$ is the LS-estimated channel for all $N_{T}\times N_{R}$ channel streams, so the number of features for the input is $2N_{T}N_{R}$. The output of the bi-LSTM network is the corresponding true channel as

(48) On−bi−LSTM=Reh^n(0);Imh^n(0),⋯,Reh^n(L−1);Imh^n(L−1),

The purpose of using a bi-LSTM network is to minimize the MSE between the predicted channel and the true channel; thus, the MSE loss function is considered. The objective function of bi-LSTM network is expressed as

(49) $$L_{\mathrm{bi}-\mathrm{LSTM}}\left(W,B\right)=\frac{1}{NL}\sum_{n=1}^{N}\sum_{i=0}^{L-1}∥{\hat{h}}^{n}\left(i\right)-h^{n}\left(i\right){∥}_{2}^{2},$$

where $h^{n}\left(i\right)$ is the true channel corresponding to ${\hat{h}}^{n}\left(i\right)$; W and B are all the weights and biases of bi-LSTM; *N* is the total number of training samples; and the superscript *n* denotes the *n*-th training sample. The loss function can be minimized by updating W and B using gradient descent algorithms. We note that this paper considers the perfect instantaneous channels to be available for the training stage, and therefore we emphasize the imperfect channel state information as a potential extension of our work in the future.

**Remark** **1.** *The deep learning-based channel estimation framework studied in this paper is based on the assumption that the perfect CSI is available during the training stage. Such information can be very accurately estimated by the orthogonal pilot signals with a sufficiently large power budget. Even though these conditions for the pilot signals increase the cost for the training stage, the neural networks can learn the channel profile properly. The effects of imperfect channels on the training of neural networks along with the performance reduction in the testing stage as a consequence are of practical interest, which will lead to solid works in the future.*

### 3.5. Computational Complexity

In this section, the complexity of the three deep learning models proposed to assist in the channel estimation phase is analyzed by utilizing big-O notation. The computational complexity of the proposed models involves two main parts: offline training and online prediction. The complexity analysis for offline training is still an open problem due to the complex implementation of the back-propagation process. However, we assume that the complexity of offline training can be afforded since it is an offline process [42]. Therefore, we only concentrate on the complexity of the online prediction phase. We use big-O notation, which is a common method to describe the complexity of the proposed deep learning-based channel estimations. The number of arithmetic operations with the dominant costs is used as the metric to obtain the computational complexity order [7].

For the FDNN-based channel estimation, from (26), we can see that if the model has *H* hidden layers, the total number of arithmetic operations has a computational complexity in the order of

(50) $$C_{\mathrm{FDNN}}=O\left(In_{1}+n_{H}K+\sum_{i=1}^{H-1}n_{i}n_{i+1}\right),$$

where *I*, *K*, and $n_{i}$ denote the input size, output size, and the number of neurons in the *i*-th hidden layer, respectively. Therefore, for one OFDM symbol, the input and output size is chosen as $I=K=2N_{T}N_{R}$, and we have $N_{FFT}$ samples. By using (50), the FDNN model has a complexity that can be shown as

(51) $$C_{\mathrm{FDNN}}=O\left(N_{FFT}\left(2N_{T}N_{R}n_{1}+2N_{T}N_{R}n_{K}+\sum_{i=1}^{H-1}n_{i}n_{i+1}\right)\right).$$

We now investigate the computational complexity of the CNN-based channel estimation. Given that there are $c_{l}$ kernels of size $k_{l}\times k_{l}$ in the *l*-th convolution layer, the number of multiplications for the *l*-th convolution layer is $k_{l}^{2}a_{l}^{1}a_{l}^{2}c_{l-1}c_{l}$, where $a_{l}^{1}$ and $a_{l}^{2}$ are sizes of the *l*-th layer. Therefore, the complexity of all convolution layers is $O(\sum_{l\in L}k_{l}^{2}a_{l}^{1}a_{l}^{2}c_{l-1}c_{l})$ [43]. The number of multiplications for the linear layer equals $O(a_{L}^{1}a_{L}^{2}c_{L}a_{linear}^{1}a_{linear}^{2})$. Since, for one OFDM symbol, the sizes of the convolution layer and the linear layer are $\left(2N_{T}N_{R}\right)\times N_{FFT}$, the total number of multiplications required in the CNN model can be calculated to be in the order of

(52) $$C_{\mathrm{CNN}}=O\left(4c_{L}{\left(N_{T}N_{R}N_{FFT}\right)}^{2}+2N_{T}N_{R}N_{FFT}\sum_{l\in L}c_{l-1}c_{l}k_{l}^{2}\right).$$

For the bi-LSTM network, it is well-known that the computational complexity of a bi-LSTM cell is $O(bi\left(4n_{i}n_{c}+4n_{c}^{2}+3n_{c}+n_{c}n_{o}\right))$ [44], where bi is the bidirectional flag (bi=2 for bi-LSTM). The notations $n_{i},n_{c}$, and $n_{o}$ denote the input size, the number of memory cells, and the output size, respectively. As mentioned before, the input and output of the bi-LSTM network include the $2N_{T}N_{R}$ features. The sequence length for one OFDM symbol can be chosen as $L=N_{FFT}$. Therefore, the computational complexity of bi-LSTM network is in the order of

(53) $$C_{\mathrm{bi}-\mathrm{LSTM}}=O\left(\left(10N_{T}N_{R}n_{c}+6n_{c}+8n_{c}^{2}\right)N_{FFT}\right).$$

## 4. Simulation Results

In this section, we evaluate the performance of the proposed deep learning-based channel estimations over the 5G channel profile and compare it with the traditional methods; i.e., LS and LMMSE. We also provide an explanation for each obtained result. First, the settings for the simulation are described, and then the simulation results for three different aspects are presented and analyzed.

### 4.1. Simulation Settings

In the simulation, we considered the MIMO-OFDM system with the parameters shown in Table 1. To model the 5G channel, we used the fading multi-path model channel with the TDL-C Power Delay Profile [33], and the 5G channel was generated using the 5G Matlab toolbox as mentioned in Section 2. The parameters used for the FDNN model, CNN model, and bi-LSTM model are given in Table 2, Table 3 and Table 4, respectively. In order to train and test the FDNN model, a set of data with 245,760 realizations was gathered. We used 70% of the data for training, 15% as the validation set, and 15% of the data for testing. For the CNN model and bi-LSTM model, we used a data set of 10,000 realizations with the same proportions for the training set, validation set, and test set as the FDNN. The parameters for training those models are shown in Table 5.

All the proposed DL-based channel estimation methods were implemented on a computer with an Intel Core i5-10400 CPU @2.90 GHz, an NVIDIA GeForce GTX 1050 Ti 16 GB memory. Matlab 2021a was used for the Monte-Carlo simulations.

### 4.2. Performance Comparison with the Conventional Estimators

To evaluate the performance of the proposed estimators, the simulation was carried out and the results compared with the conventional LS estimation and LMMSE estimation by utilizing the bit error rate (BER) and mean square error (MSE) versus signal to noise ratio (SNR).

To investigate the performance of all the considered channel estimations used in the MIMO-OFDM system through the 5G channel model, two different scenarios corresponding to the velocity of mobiles were exploited. In the first scenario, the receiver moved with a low speed such that the maximum Doppler frequency was 36 Hz. The pilot symbols were inserted along with data in both frequency and time domains. In the frequency domain, we referred to the type 1 configuration of DM-RS as in [36]. In this configuration, six subcarriers were defined for the DM-RS signal for each physical resource block that contained 12 subcarriers. Thus, the pilot spacing in the frequency domain was $D_{f}=2$ for both scenarios. In the time domain, the 5G system supported up to 4 pilot symbols in 1 slot that included 14 OFDM symbols. Therefore, in the first scenario, since the channel slowly changed over time, the pilot spacing in the time domain was $D_{t}=14$. In the second scenario, the system exhibited high-speed mobility, which resulted in the maximum Doppler frequency of 200 Hz. In this scenario, the setup $D_{t}=7$ was used to cope with the rapid change of the channels over time.

**Figure 9:**
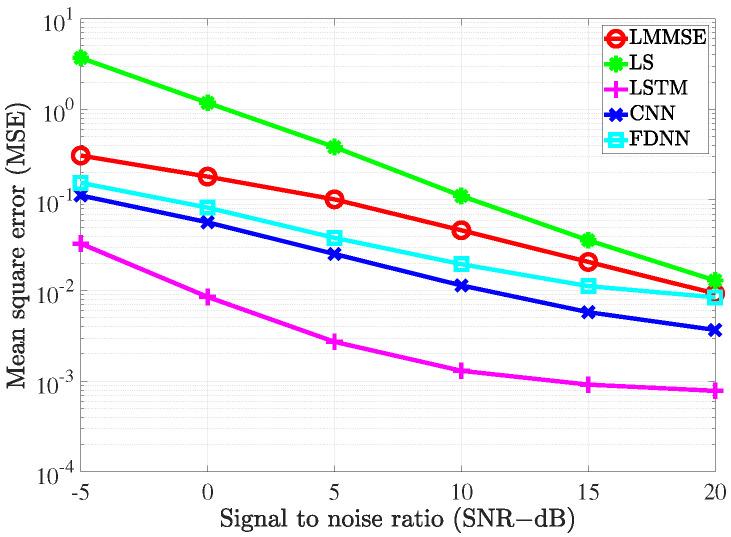
The MSE of the channel estimate vs. the SNR level with $f_{D}=36$ Hz.

**Figure 10:**
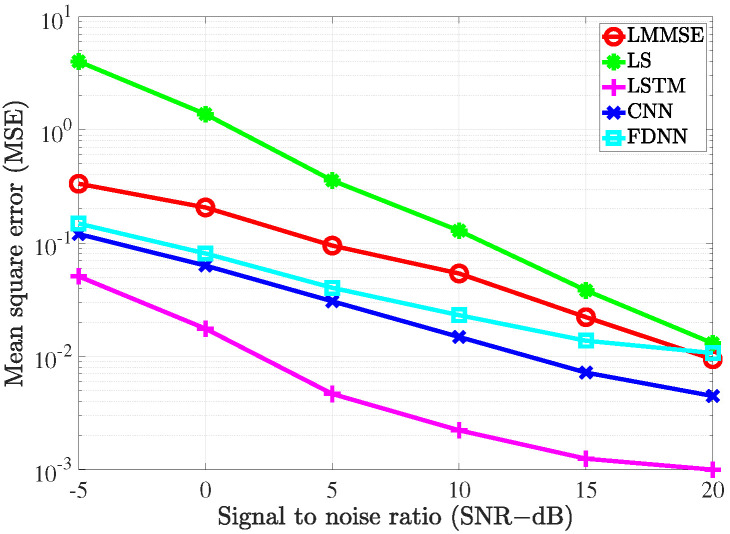
The MSE of the channel estimate vs. the SNR level with $f_{D}=200$ Hz.

**Figure 11:**
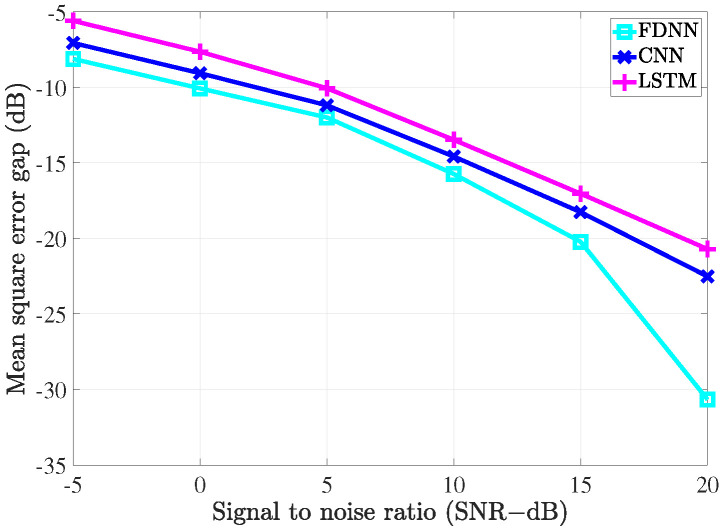
The MSE gap (dB) between the deep learning-based channel estimation methods and the LMMSE estimation with $f_{D}=36$ Hz.

**Figure 12:**
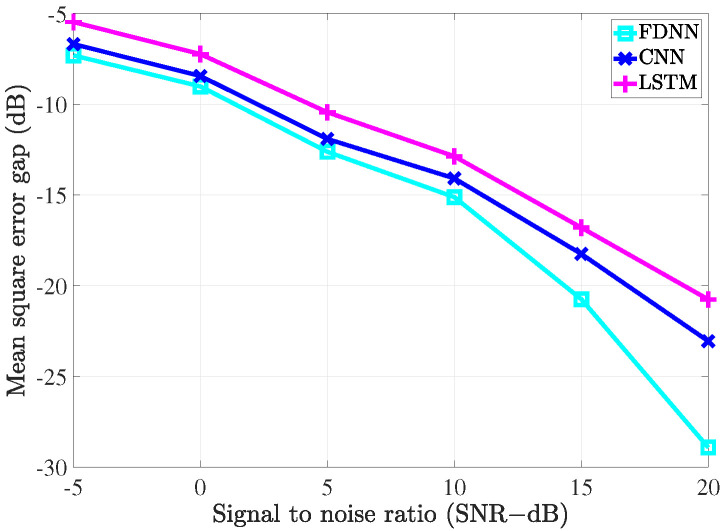
The MSE gap (dB) between the deep learning-based channel estimation methods and the LMMSE estimation with $f_{D}=200$ Hz.

Figure 9 and Figure 10 show the MSE of different channel estimations in the first and second scenarios, respectively. The 16-QAM (quadrature amplitude modulation) method was deployed to modulate the transmitted data in the simulation. As shown in Figure 9 and Figure 10, all the channel estimation methods led to the MSE declining gradually as the SNR increased. In both the scenarios, LS estimation yielded the worst MSE performance, which was because it does not take the statistical channel information into account when performing the channel estimation. On the contrary, LMMSE estimation exploits the mean and covariance matrices, which resulted in better MSE performance than its LS counterpart. Our proposed deep learning estimators yielded the best MSE performance compared to the two conventional methods. In detail, the FDNN model showed the smallest MSE compared to the two other deep learning models. This is because the FDNN model has the simplest structure; thus, it could not study the structure of the channel as well as the others. The CNN model, on the other hand, not only could learn more deeply than the FDNN model but also provided robustness in denoising noisy data. Therefore, we can see that the CNN model yields better performance compared to the FDNN model. However, both FDNN and CNN models could not exploit the relation between channels in the same way as the bi-LSTM model. Therefore, we can see a great improvement in terms ofMSE performance due to the bi-LSTM model. To further clarify this, the MSE gaps (dB) between the deep learning-based channel estimation methods and the LMMSE estimation are shown in Figure 11 and Figure 12. From the two figures, it can be seen that the gaps decrease as the SNR level increases. Thus, the deep learning-assisted methods work much better in the low SNR region. Comparing between the two scenarios, due to the change of the pilot density, the performance differences between two scenarios are not significant.

**Figure 13:**
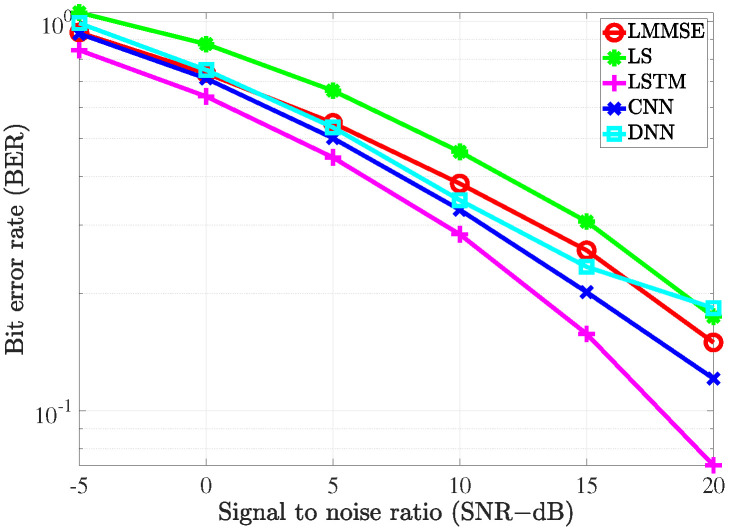
The BER of the channel estimate vs. the SNR level with $f_{D}=36$ Hz.

**Figure 14:**
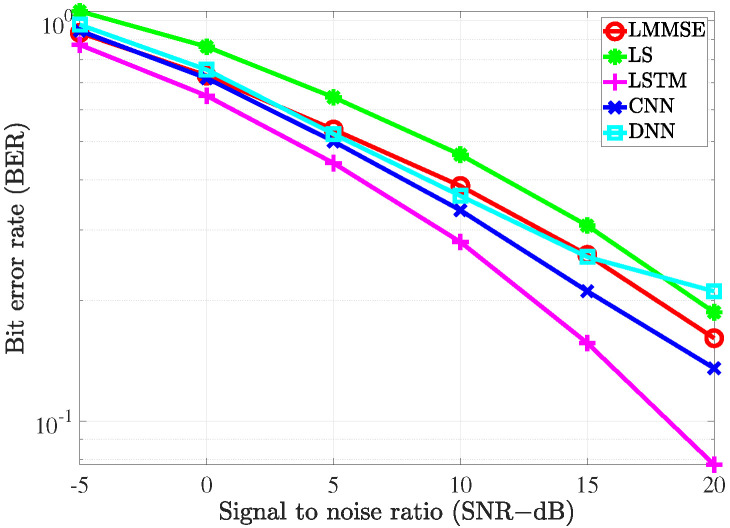
The BER of the channel estimate vs. the SNR level with $f_{D}=200$ Hz.

We also provide the BER performance of the considered scenarios in Figure 13 and Figure 14 with the different channel estimation methods, respectively. The trend of the BER performance for the examined estimators is similar to that of MSE performance. However, in both scenarios, the BER performance of the FDNN model is slightly worse than the LMMSE method at SNR=20 dB. This can be explained by the fact that the loss function has been defined to minimize the channel estimation errors instead of the BER metric.

### 4.3. System Performance versus Pilot Density

**Figure 15:**
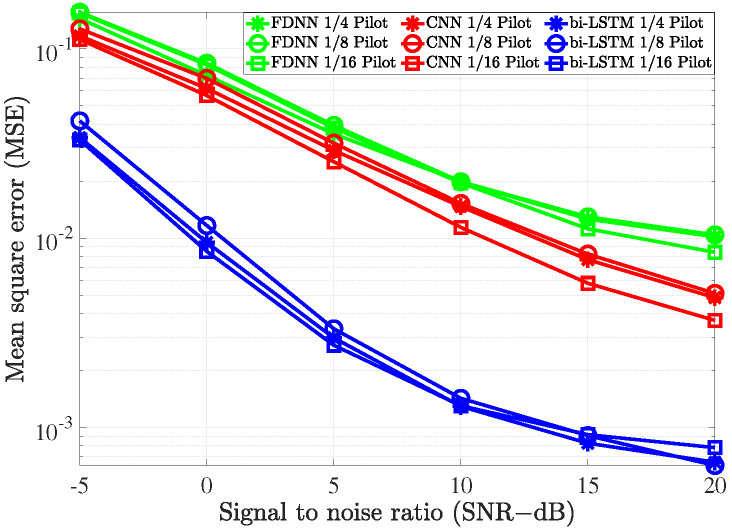
The impact of the pilot density on the deep learning-based channel estimations.

The impact of pilot density is illustrated in Figure 15 to evaluate the robustness of deep learning estimators. As the pilot density decreased, the performance of the three deep learning estimators remained unchanged with different values of SNR. Thus, we can conclude that the deep learning estimation models are robust to different pilot densities.

### 4.4. System Performance versus Maximum Doppler Frequency

**Figure 16:**
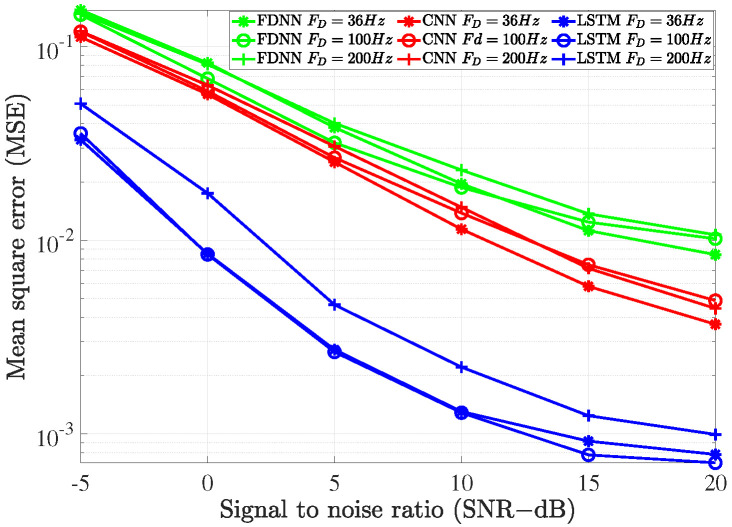
The impact of the Doppler frequency on the deep learning-based channel estimations.

In this subsection, we evaluate the influence of the maximum Doppler frequency $f_{D}$ on the proposed deep learning models. As shown in Figure 16, when the maximum Doppler frequency increased, the performance of the deep learning models decreased. This can be explained by the fact that the channel varied faster as the Doppler frequency increased. From the figure, we also see that the performance of bi-LSTM model decreased more severely compared to the others. However, its performance was still significantly better than that of FDNN and CNN models.

**Figure 17:**
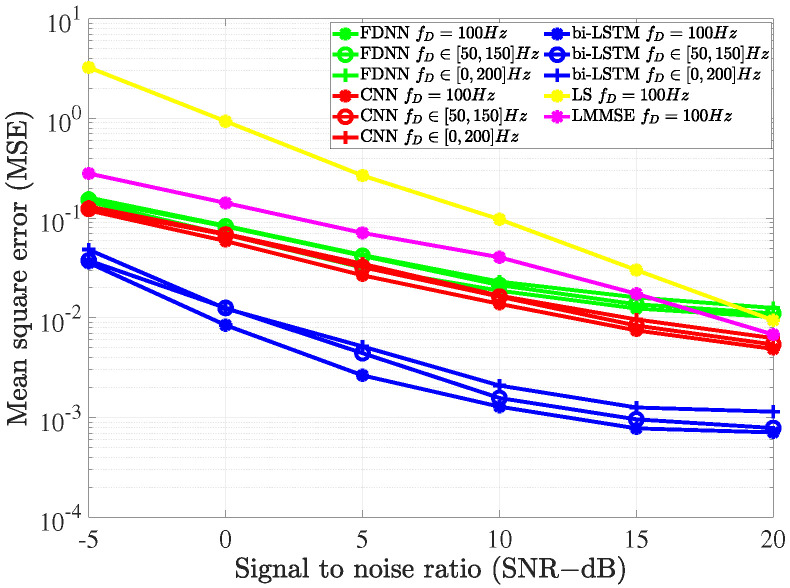
The impact of a Doppler frequency mismatch on the deep learning-based channel estimations.

In practice, to investigate the sensitivity of the neural networks, we considered a scenario in which the Doppler frequency $f_{D}$ varied constantly due to the change of the receiver’s velocity. Alternatively, we evaluated the prediction accuracy of the proposed models when there was a mismatch of the Doppler frequency between the training stage and testing stage. In this simulation, we kept the value fo $f_{D}=100$ Hz in the training stage, while the value of $f_{D}$ in the testing stage was randomly distributed from a uniform distribution. The result is illustrated in Figure 17. As seen from the figure, all the deep-learning channel estimation models were robust to the mismatch of the Doppler frequency. Only the performance of the bi-LSTM model decreased slightly when SNR=20 dB. The performance of the DNN models in the different cases was also compared to the LS and LMMSE estimation methods when $f_{D}=100$ Hz. All the deep learning-based channel estimation models outperformed the conventional models, even in the case of channel mismatching. From the observations, we conclude that the bi-LSTM model is more sensitive to the Doppler frequency compared to the FDNN and CNN models. The reason is that the bi-LSTM model exploits the time-varying properties of channels; thus, the Doppler frequency has more serious effects on the bi-LSTM model. However, the three proposed models are still robust to the changes of the Doppler frequency and therefore more efficient than the conventional methods.

## 5. Conclusions

In this paper, we have presented the use of different DNN structures, including a fully-connected DNN, CNN, and bi-LTSM, to assist in the channel estimation process in a MIMO-OFDM system with different scenarios of fading multi-path channel models based on the TDL-C model defined in the 5G networks. The proposed DNN-based channel estimation framework was trained with the channel estimation from least squares estimation and the corresponding perfect channels to obtain the parameters as weights and biases. By utilizing the QAM modulation scheme, the performance of the proposed estimations was compared with the conventional LS and LMMSE estimations in terms of the channel estimation error and the bit error ratio as a function of the SNR levels. As the channel properties were learned effectively, we observed improvements of the proposed deep learning-aided estimations in terms of reducing the channel estimation error and bit error ratio. Among the proposed deep learning-based channel estimation approaches, bi-LSTM showed the greatest reduction in channel estimation error as a consequence of its ability to exploit the time and frequency correlation among the channels. Furthermore, the proposed deep learning-based channel estimation approaches exhibited great robustness with different pilot densities as well as with changes of the Doppler frequency.

## Figures and Tables

**Table 1:** The parameter setup for the considered MIMO-OFDM system.

Parameters	Values
MIMO	4 × 4
FFT size	256
Subcarrier spacing	15 kHz
Cyclic prefix	24
Type of modulation	16-QAM
Channel PDP	TDL-C
Maximum Doppler frequency	36 Hz, 200 Hz
Noise model	Gaussian Noise
Sample frequency	3.84 MHz

**Table 2:** The architecture setup of the FDNN-based channel estimation.

Layer	Nodes	f(.)
Input layer	32	-
Hidden layer 1	64	tanh
Hidden layer 2	64	tanh
Hidden layer 3	64	tanh
Output layer	32	-

**Table 3:** Architecture of CNN model for channel estimation.

Layer	Kernel	f(.)
Input layer	16 × 256	-
Conv1 layer	3 × 3 × 64	ReLU
Conv2 layer	3 × 3 × 64	ReLU
Conv3 layer	3 × 3 × 64	ReLU
Conv4 layer	3 × 3 × 32	ReLU
Linear layer	-	-

**Table 4:** Architecture of bi-LSTM model for channel estimation.

Parameter	Value
Number of input feature layers	32
Number of LSTM layers	2
Hidden layer size	100
Sequence length	256
Activation function	Tanh and Sigmoid

**Table 5:** Parameters for training deep learning models.

Parameters	Values
Optimizer	Adam
Maximum number of epoches	100
Mini-bath size	32
Training error	$10^{-5}$
Gradient descent accuracy	$10^{-7}$
Learning rate	0.001
Maximum validation failures	6

## Data Availability

The data for the simulation in this paper including noisy channel and theory channel can be found in this repository: https://drive.google.com/drive/folders/1KWCS9Yc3jh-IEkXs7rbjR8GW8e4uOzj4?usp=sharing accessed on 15 July 2021.

## Abbreviations

The following abbreviations are used in this manuscript:

5G	Fifth generation
3GPP	Third generation partnership project
BER	Bit error ratio
CNN	Convolutional neural network
CP	Cyclic prefix
DNN	Deep neural network
FDNN	Fully connected deep neural network
FFT	Fast Fourier transform
ISI	Inter-symbol interference
LMMSE	Linear minimum mean square error
LS	Least Squares
LSTM	Long short-term memory
GRU	Gated recurrent unit
MIMO	Multiple-input multiple-output
MSE	Mean square error
OFDM	Orthogonal frequency-division multiplexing
QAM	Quadrature amplitude modulation
SNR	Signal to noise ratio
TDL-C	Tapped delay line type C model
